# A Multi-Module Fusion Framework for Restoring Human and Machine Vision Quality in Compressed Video

**DOI:** 10.3390/s26113494

**Published:** 2026-06-01

**Authors:** Keren He, Kun Xiang, Yufei Gao, Yang Yu, Jinjia Zhou

**Affiliations:** Graduate School of Science and Engineering, Hosei University, Tokyo 184-8584, Japan; keren.he.6r@stu.hosei.ac.jp (K.H.); kun.xiang.68@hosei.ac.jp (K.X.); yufei.gao.8e@stu.hosei.ac.jp (Y.G.); yang.yu.3m@stu.hosei.ac.jp (Y.Y.)

**Keywords:** video coding for machines (VCM), post-processing, object detection, object segmentation

## Abstract

With the increasing demand for video processing in both human perception and machine vision applications, enhancing heavily compressed video has become a critical problem in practical multimedia systems. In many real-world scenarios, video data acquired by image sensors are often compressed for efficient transmission and storage, which introduces compression artifacts and degrades both visual quality and downstream task performance. This issue is especially significant in sensor-based systems such as surveillance cameras and mobile imaging devices. To address these challenges, we propose a novel joint human–machine video enhancement framework for compressed video enhancement that jointly targets human perceptual quality and machine vision performance. The framework integrates four complementary components: a Spatio-Temporal Fusion Module that leverages inter-frame correlations, a High-Frequency Semantic Fusion module for recovering structurally important details relevant to machine tasks, a Texture-Guided Model that enhances low-level visual features, and a Refined Attention Residual Quality Enhancement Module that adaptively emphasizes salient regions. By progressively combining these modules, the framework effectively restores compressed content while preserving task-relevant semantics. The experimental results demonstrate that our method consistently outperforms existing approaches, achieving higher PSNR and SSIM as well as improved object detection and video object segmentation performance. These results highlight the framework’s practical applicability for compressed video enhancement in sensor-based systems, including intelligent surveillance and autonomous imaging platforms.

## 1. Introduction

In recent years, deep learning has greatly advanced computer vision technology. Computer vision techniques are now widely used in many real-world applications, including medical image analysis, autonomous driving, video surveillance, and augmented reality [1]. However, many of these applications rely on compressed and decoded images or video streams as input. Distortions introduced by image and video compression not only degrade perceptual quality for human viewing but also reduce the reliability of downstream machine vision systems operating on compressed visual content. Existing video codecs such as High Efficiency Video Coding (HEVC) [2] and Versatile Video Coding (VVC) [3] are mainly designed for human visual perception and primarily optimize perceptual fidelity for human observers. Consequently, they are not fully suitable for downstream machine vision tasks such as image classification, object detection, and semantic segmentation. To address this issue, the Moving Picture Experts Group introduced the concept of “Video Coding for Machines (VCM)” [4], aiming to jointly consider compression efficiency and downstream machine analysis performance.

Recent advances in machine-oriented compression and feature coding have demonstrated remarkable efficiency in downstream machine vision tasks. Existing methods mainly preserve task-relevant semantic information through feature-level compression, semantic-aware coding, and bitrate allocation strategies [5,6,7,8,9,10,11,12,13,14]. Gao et al. [5] studied how to make video compression better for machine use and proposed a method to combine compression with intelligent processing. Recent studies [10,11,12] have proposed methods for feature-level enhancement of video compression for machine vision. Related work [6,7,8] proposed some feature compression-based methods that can jointly optimize the compression rate and the task objective of interest. The study in [6] reorganized the feature data into video input and used HEVC for compression, while [7,8] used neural networks to compress the input features. This study in [9] proposed a video feature compression system based on latent variable representation to support downstream machine reasoning tasks such as object detection and instance segmentation. More recently, machine-oriented image and video compression methods based on Just Recognizable Distortion (JRD) and machine vision redundancy (MVR) [13,14] further demonstrated that machine perception mainly relies on task-relevant semantic structures rather than global perceptual fidelity. These methods improve coding efficiency through semantic-aware bitrate allocation and machine-perceived redundancy suppression for downstream vision tasks. Although these machine-oriented compression methods are effective in preserving task-relevant semantic information for downstream vision tasks, they mainly focus on coding efficiency and semantic-aware bitrate allocation during compression. As a result, they often provide limited support for perceptual reconstruction quality for human observers. Strong compression and selective semantic preservation may cause permanent loss of important visual details such as textures, colors, and edge sharpness. Consequently, the reconstructed video may appear blurry, artifact-prone, and visually unnatural, especially under low-bitrate conditions, where only task-critical information is preserved for machine understanding rather than perceptual quality restoration.

However, these observations indicate that optimizing compressed visual content solely for machine perception is insufficient for many real-world multimedia systems. In practical scenarios such as intelligent surveillance, autonomous driving, and sensor-based video analytics, compressed visual content is often simultaneously consumed by both human observers and machine vision systems. Consequently, recent research has gradually shifted from conventional human-oriented or machine-oriented visual optimization toward human–machine collaborative multimedia processing, where perceptual reconstruction quality and semantic representation quality are jointly optimized for both human observers and machine vision systems [15]. Recent studies have extensively explored joint human–machine visual optimization for compressed image and video processing [16,17,18,19,20]. Existing approaches generally attempt to establish unified representations that simultaneously support human perceptual reconstruction and downstream machine vision tasks. Early collaborative frameworks mainly relied on scalable coding and separate optimization pipelines for human and machine vision [18], which often introduced considerable bitrate redundancy and computational overhead. Subsequent works further investigated rate-distortion optimization for machine-oriented visual analysis [19], highlighting the fundamental inconsistency between perceptual fidelity and machine-oriented semantic representation. More recent methods attempted to improve compression efficiency through unified and compact feature representations shared across multiple tasks [16,17]. In particular, unified human–machine coding frameworks explored multi-path aggregation, conditional reconstruction, and task-oriented modulation strategies to jointly support perceptual reconstruction and downstream machine analysis. Furthermore, implicit neural representation-based collaborative compression methods [20] further demonstrated the potential of jointly optimizing perceptual quality and machine vision utility within a unified framework. Although these methods achieve promising performance for collaborative visual processing, they still suffer from the inherent difficulty of multi-objective optimization between perceptual fidelity and semantic representation quality.

To alleviate these limitations, we investigate the problem from the perspective of post-compression enhancement rather than collaborative encoding optimization. Compared with encoding-side collaborative compression frameworks, post-processing-based enhancement methods provide greater flexibility for practical deployment because they can be directly integrated into existing compression and transmission pipelines without redesigning the codec architecture. More importantly, enhancement-based frameworks enable explicit restoration of texture details, high-frequency semantic structures, and task-oriented representations after compression, making them more suitable for jointly improving perceptual reconstruction quality and downstream machine vision performance.

Existing compressed video enhancement and post-processing methods [21,22,23,24,25,26,27] mainly optimize pixel-level reconstruction quality using perceptual objectives such as PSNR and SSIM. Recent methods [28,29] further attempted to improve robustness across different compression levels. However, these enhancement-based approaches still mainly focus on perceptual reconstruction quality while lacking sufficient modeling of semantic-frequency structures and task-oriented representations, required by downstream machine vision tasks under heavy-compression conditions. Therefore, maintaining downstream machine vision performance while preserving human visual quality remains a key challenge in human–machine collaborative multimedia processing and compressed video restoration [30]. To address this challenge, we propose an end-to-end framework for machine-perception-friendly compressed video restoration, which jointly models temporal consistency, semantic-frequency structures, texture-aware restoration, and task-oriented feature representations under compressed video conditions.

The overall pipeline begins by fusing spatio-temporal features of compressed video sequences. Specifically, we adopt only the deformable spatio-temporal fusion mechanism from STDF [27] as a lightweight temporal alignment backbone to aggregate neighboring frame information, while the subsequent enhancement pipeline is fully redesigned for joint human–machine vision optimization. The proposed framework further incorporates a High-Frequency Semantic Fusion (HFSF) module, presented in our previous work [31], for preserving semantic high-frequency structures and a novel Texture-Guided Model (TGM), which explicitly decomposes compressed representations into edge-aware structures, low-frequency stable components, and adaptive mid-frequency texture features through the combination of fixed Gaussian filtering and learnable dynamic convolution. This design enables the framework to effectively recover compression-sensitive texture details while maintaining structural consistency.

After texture-guided enhancement, the fused representation is further refined by the proposed RAR-QE module. Building upon this, the Refined Attention Residual Module introduces channel-adaptive weighting and spatial-aware attention into residual feature refinement, enabling the framework to suppress compression artifacts while simultaneously preserving semantic structures useful for downstream detection and segmentation tasks. By integrating temporal fusion, semantic-frequency enhancement, texture-guided restoration, and adaptive residual refinement into a unified framework, the proposed method simultaneously improves perceptual reconstruction quality and downstream machine vision performance under compressed video conditions. The main contributions of this paper can be summarized as follows:

- We propose a unified compressed video enhancement framework that jointly considers human visual perception and downstream machine vision tasks. Different from conventional restoration-oriented methods, the proposed framework explicitly enhances machine-friendly feature representations while maintaining perceptual reconstruction quality under compressed video conditions.
- We redesign the enhancement pipeline based on semantic-frequency-aware restoration rather than conventional pixel-level refinement. Specifically, only the deformable spatio-temporal fusion mechanism from STDF is adopted as a lightweight temporal alignment module, while the subsequent enhancement framework is newly developed for joint human–machine optimization.
- We propose a novel Texture-Guided Model (TGM) for compression-aware texture restoration. The proposed TGM explicitly decomposes compressed representations into edge-aware structures, low-frequency stable components, and adaptive mid-frequency texture features through the combination of fixed Gaussian filtering and learnable dynamic convolution, enabling more effective recovery of compression-sensitive structural details.
- We further propose a Refined Attention Residual Quality Enhancement (RAR-QE) module, which introduces channel-adaptive weighting and spatial-aware residual attention to suppress compression artifacts while preserving semantic structures beneficial for downstream detection and segmentation tasks.

## 2. Related Works

### 2.1. Image/Video Compression for Machine Vision

With the development of deep learning, many researchers have begun to explore the connection between compression and downstream tasks.

Some studies [18,30,32,33,34,35,36,37] also focus on the joint optimization of image compression and downstream machine vision tasks. For example, Lu et al. [30] use an image preprocessing method to minimize the distortion for machine vision tasks such as image classification and object detection. In [32], a CNN post-processing filter is used to enhance the image quality. These methods have shown success in machine vision tasks, but they focus solely on images and fail to handle video-specific challenges like temporal dependencies and motion features. Our work addresses this gap by developing a video-oriented approach.

Recent studies [6,7,8,10,11,12] have introduced various approaches to improve video compression at the feature level to improve machine vision performance. Among these, several works have explored feature compression techniques that jointly optimize compression efficiency and task-specific objectives. For example, the work in [6] reorganized feature data into video-like inputs and leveraged standard video codecs such as HEVC for compression. The work in [9] employed latent variable representations to compress video features in a way that preserved their utility for downstream machine tasks.

While some methods can achieve significant performance gains in machine vision tasks, they may not yield comparable improvements in terms of human visual experience. For instance, the approach proposed in [4] employs a neural network to enhance image recognition accuracy through post-processing of the encoded video, utilizing features from YOLOv7 during training. Although this method improves object detection accuracy, it might not fully restore the visual quality degradation introduced by the post-processing stages.

### 2.2. Compressed Video Enhancement for Human Vision

Existing compressed video enhancement methods can be divided into in-loop methods and post-processing methods. Post-processing methods [21,22,23,24,25,26,27,29,38,39,40,41,42,43,44,45,46] provide more practical solutions for compressed video enhancement.

MFQE [22] developed an SVM-based peak quality frame (PQF) detector for video enhancement, analyzing spatiotemporal features to identify high-quality frames and enhance neighboring frames. While effective for human vision, its performance on machine vision tasks (e.g., object detection) is limited due to SVM’s inability to capture high-level semantic features and potential artifact generation. MFQE 2.0 [23] introduced a BiLSTM-based detector to better model temporal dependencies, but it faces challenges in computational efficiency. Moreover, its enhancement process does not necessarily preserve features critical for machine vision applications, like action recognition. STDF [24] proposed spatio-temporal deformable convolution to address optical flow inaccuracies in compressed videos, learning dynamic offset fields instead of relying on traditional flow estimation. However, its pixel-level optimization may not align with higher-level vision tasks requiring semantic understanding. Building upon these insights, we adapt STDF’s deformable convolution as our core module while enhancing its feature representation capabilities specifically for machine vision applications.

## 3. Proposed Method

### 3.1. Overview

The proposed framework takes decoded compressed video frames as input and consists of four main components: a Spatio-Temporal Fusion module, a High-Frequency Semantic Fusion (HFSF) module, a Texture-Guided Module (TGM), and a Refined Attention Residual Quality Enhancement (RAR-QE) module. The enhanced outputs are then used for both perceptual reconstruction and downstream machine vision tasks.

**Figure 1:**
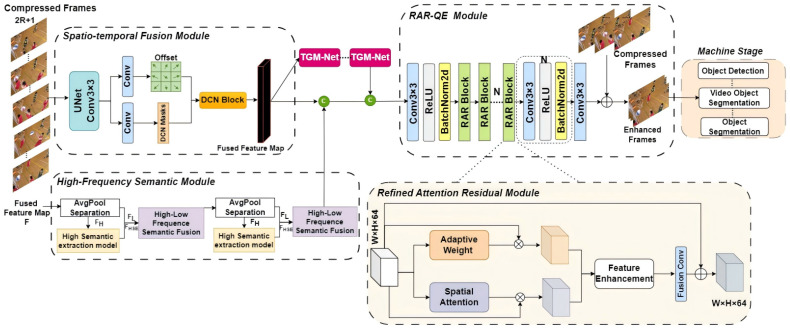
Overview of our proposed modules.

Figure 1 demonstrates the overall framework of our method. Given a compressed frame $I_{t}$ and its temporal neighbors $\left\{I_{t-k},\ldots ,I_{t+k}\right\}$, our model outputs an enhanced frame ${\hat{I}}_{t}$ and intermediate features that are friendlier to downstream tasks (e.g., detection, segmentation). A lightweight U-Net backbone and deformable convolutions are first used to align and fuse spatio-temporal features. Then, the Texture-Guided Modules (TGM-Nets) enhance textures and details using edge- and frequency-guided methods. Subsequently, the RAR-QE module decomposes and fuses multi-frequency features via learnable convolutions, while the Refined Attention Residual Module performs channel- and spatial-aware feature enhancement in a residual manner, suppressing artifacts while preserving structural cues. The enhanced output improves visual quality and machine task performance. Since all components are convolutional, this framework can be jointly optimized with upstream or downstream tasks in an end-to-end manner.

### 3.2. Basic Fusion Modules

Spatio-Temporal Deformable Fusion Module: To exploit neighboring frames under compression noise and motion, we use a Basic STDF Fusion module [24] to fuse contextual information by spatio-temporal deformable convolution using the target and reference frames as input. For each neighbor $X_{\tau }$, we predict sampling offsets $\Delta_{\tau }$ and modulation masks $m_{\tau }$ via a light offset network A(·) and apply deformable convolution D(·):

(1) $${\tilde{X}}_{\tau \;\to t}=D\;\left(X_{\tau };\;\Delta_{\tau },m_{\tau }\right),\;\left[\Delta_{\tau },m_{\tau }\right]=A\;\left([X_{\tau },X_{t}]\right).$$

Aligned features $\left\{{\tilde{X}}_{\tau \;\to t}\right\}$ are concatenated with the reference $X_{t}$ and fused through a shallow U-Net/Conv block to obtain the fused feature map

(2) $$F_{t}=F\left([X_{t},{\tilde{X}}_{t-k\;\to t},\ldots ,{\tilde{X}}_{t+k\;\to t}]\right).$$

High-Frequency Semantic Fusion Module: Using the High-Frequency Semantic Fusion (HFSF) module designed in our previous work [31], we extract and enhance high-frequency features while preserving the integrity of the original information. Subsequently, the enhanced high-frequency features are concatenated with the basic fused feature map $F_{t}$ to fully integrate multi-scale and multi-level feature information:

(3) $$F_{\mathrm{HF}}=\mathrm{HFSF}\left(F_{\mathrm{in}}\right).$$

(4) $$F_{\mathrm{Fused}}=\mathrm{Concat}\left(F_{t},F_{\mathrm{HF}}\right).$$

where $F_{\mathrm{in}}$ is the input feature map, and $F_{\mathrm{HF}}$ represents the enhanced high-frequency features.

### 3.3. Texture-Guided Model

**Figure 2:**
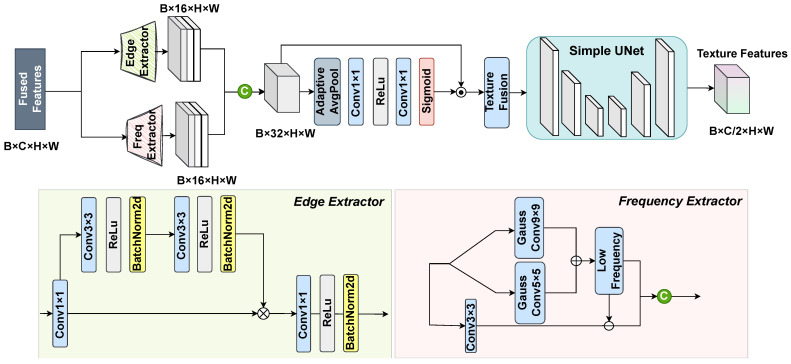
Overview of the proposed Texture-Guided Model (TGM).

Figure 2 illustrates the overall architecture. Given an input feature map $F_{\mathrm{in}}$, the proposed Texture-Guided Model (TGM) is designed to recover texture information degraded by compression by explicitly modeling complementary edge-aware and frequency-aware cues. The input $F_{\mathrm{in}}$ is processed in parallel by an edge extractor and frequency extractor. Their outputs are concatenated along channels, reweighted by SE-style channel attention, fused with two 3 × 3 convolutions, and finally refined by a lightweight U-Net to yield $F_{\mathrm{tex}}$. Different from purely data-driven refinement blocks, TGM introduces an explicit texture decomposition strategy, where stable low-frequency structures and compression-sensitive mid/high-frequency details are modeled separately. By introducing texture-sensitive representations into the enhancement pipeline, TGM provides complementary structural details that are beneficial to both perceptual restoration and downstream machine vision tasks.

Edge extractor: The edge extractor is designed to emphasize contour-sensitive and high-frequency structures that are often weakened by compression artifacts. Since accurate boundary representation is important for downstream tasks such as object detection and segmentation, this branch aims to preserve spatial detail at relatively high resolution. To achieve this, the branch adopts a lightweight convolutional design that combines channel reduction and residual feature transformation, enabling edge-aware feature extraction with low computational overhead. Given the input feature map X, the edge branch produces

(5) $$\begin{array}{cc} F_{\mathrm{edge}} & =\left(\left(\mathrm{ReLU}\;\circ \;\mathrm{BN}\;\circ \;{\mathrm{Conv}}_{3\times 3,\;1,\;1}^{C\to C_{e}}\right)\left(X\right)\right)\\ \; & \;⊕{\mathrm{Conv}}_{1\times 1,\;1,\;0}^{C\to C_{e}}\left(X\right)\in R^{B\times C_{e}\times H\times W}. \end{array}$$

where $C_{e}=16$, and ⊕ denotes element-wise addition. This branch highlights edge-aware responses and preserves contour-related structures that are useful for both texture restoration and task-oriented feature enhancement.

Frequency extractor: The frequency extractor is designed to explicitly decompose texture information into complementary spectral components, so as to better recover details degraded by compression. The frequency branch combines *fixed* Gaussian blurs for low-frequency extraction and a *learnable* dynamic convolution for adaptive texture modeling. The use of fixed Gaussian filters is intentional rather than heuristic. In compressed video, low-frequency structures usually contain relatively stable spatial layout and coarse object information, while compression artifacts mainly disturb high-frequency details and boundary regions. Therefore, deterministic Gaussian low-pass filters provide a stable and noise-robust structural prior for low-frequency extraction. Specifically, fixed Gaussian filters are used to provide stable low-frequency responses, while a learnable convolution captures adaptive mid-frequency variations. Compared with fully learnable frequency decomposition, this hybrid design reduces the risk of frequency leakage and prevents the network from redundantly learning basic smoothing operations from noisy compressed inputs. As a result, the learnable convolution branch can focus more effectively on modeling compression-dependent mid-frequency texture variations. This design allows the module to preserve coarse structural information while emphasizing compression-sensitive texture details. Let $C_{f}=16$, with half devoted to low frequency and half to mid-frequency:

(6) $$\begin{array}{cc} {\mathrm{Low}}_{5\times 5} & =G_{5}*F_{\mathrm{in}},\;{\mathrm{Low}}_{9\times 9}=G_{9}*F_{\mathrm{in}}, \end{array}$$

(7) $$\begin{array}{cc} \mathrm{Dyn} & ={\mathrm{Conv}}_{3\times 3}\left(F_{\mathrm{in}}\right), \end{array}$$

(8) $$\begin{array}{cc} \mathrm{Mid} & =\mathrm{Dyn}-\left({\mathrm{Low}}_{5\times 5}+{\mathrm{Low}}_{9\times 9}\right), \end{array}$$

where $G_{5},G_{9}$ are fixed Gaussian kernels (non-trainable). Here, the two Gaussian kernels with different receptive fields provide multi-scale low-frequency references, while the subtraction operation encourages the branch to isolate residual frequency components that are more sensitive to compression-induced texture degradation. The final frequency feature is the channel-wise concatenation

$$F_{\mathrm{freq}}\;=\;\mathrm{Concat}\;\left[{\mathrm{Low}}_{5\times 5},\;{\mathrm{Low}}_{9\times 9},\;\mathrm{Mid}\right].$$

In this way, the frequency branch explicitly isolates informative mid-frequency texture while retaining stable low-frequency context. This fixed-and-learnable decomposition makes the TGM more robust under different compression levels and provides a clearer functional separation between structural preservation and texture compensation.

Texture fusion: The edge-aware and frequency-aware features provide complementary texture cues and are therefore fused in an adaptive manner. We first concatenate the two branches as

$$F_{⊕}=\mathrm{Concat}\left[F_{\mathrm{edge}},F_{\mathrm{freq}}\right]\in R^{B\times (C_{e}+C_{f})\times H\times W},$$

and then apply SE-style channel attention to emphasize informative feature channels. Here, GAP is global average pooling, σ is the sigmoid activation, and ⊙ denotes channel-wise scaling. The attention weights and reweighted feature are defined as

(9) $$\begin{array}{cc} \alpha & =\sigma \left({\mathrm{Conv}}_{1\times 1}\left(\mathrm{ReLU}\left({\mathrm{Conv}}_{1\times 1}\left(\mathrm{GAP}(F_{⊕})\right)\right)\right)\right), \end{array}$$

(10) $$\begin{array}{cc} \tilde{F} & =\alpha ⊙F_{⊕}. \end{array}$$

Two subsequent 3×3 convolutional blocks with BN and ReLU are then used to fuse and refine the attended texture representation before the final U-Net refinement stage. Through this adaptive fusion, the model can selectively emphasize the most informative edge and frequency channels according to the degradation characteristics of the compressed input.

Lightweight U-Net refinement: To further suppress artifacts and improve spatial coherence, a lightweight U-Net is applied to the fused texture representation and outputs the final texture feature. Its encoder–decoder structure, together with skip connections, progressively refines local details while preserving global structural consistency. The final output is a high-fidelity texture feature map:

$$F_{\mathrm{tex}}\in R^{B\times C_{t}\times H\times W}.$$

This texture-enhanced feature map is subsequently used as a complementary representation for quality enhancement and downstream task-oriented refinement.

### 3.4. RAR–QE Module

Video compression inevitably introduces various distortions, including block artifacts, blurring, and edge degradation. Conventional quality enhancement (QE) modules are typically designed as decoder-side post-processing components that mainly focus on spatial restoration, while paying limited attention to the semantic structures that are important for downstream machine vision tasks.

This limitation becomes more critical in compressed video understanding, where the enhancement stage directly affects not only human perceptual quality but also the reliability of subsequent detection and segmentation models. Therefore, an effective QE module should simultaneously suppress compression artifacts and preserve task-relevant structural information.

To address this issue, we propose the Refined Attention Residual Quality Enhancement (RAR-QE) module, which serves as the final adaptive refinement stage of the proposed framework. Given the fused feature representation produced by the preceding modules, RAR-QE performs structured quality enhancement to improve perceptual fidelity while strengthening semantic cues that are useful for downstream analysis. The core of this module is the proposed Refined Attention Residual Module (RARM), which combines residual learning with channel-aware and spatial-aware feature modulation.

### 3.5. Refined Attention Residual Module (RARM)

To improve feature restoration under compression, we introduce the Refined Attention Residual Module (RARM), which adopts a multi-branch residual attention design for adaptive feature refinement. Given an input feature map F, the module consists of three components: a spatial attention branch, an adaptive weight branch, and a feature enhancement branch. These components collaboratively enhance the discriminability and reconstruction quality of compressed feature representations.

Spatial attention branch: The spatial attention branch is designed to emphasize structure-sensitive spatial responses in compressed frames. It models spatial attention using both average and max pooling across the channel dimension, followed by a convolution operation. The result $A_{sp}$ is the learned spatial attention map:

(11) $$F_{sp}=A_{sp}⊗F,$$

where ⊗ denotes element-wise multiplication.

Adaptive weight branch: The adaptive weight branch estimates channel-wise importance weights to control the enhancement strength of different feature channels. The resulting weights $W_{adp}\in R^{64}$ are used to rescale the input feature channels:

(12) $$F_{adp}=F⊗W_{adp}.$$

This branch allows the module to adaptively emphasize informative channels that are more relevant to artifact suppression and semantic preservation.

Feature enhancement branch: The outputs of the two attention branches are first combined and then refined by a feature enhancement branch for residual restoration. Finally, a residual connection is introduced to integrate the original input with the enhanced features:

(13) $$F_{enh}={\mathrm{Feature}}_{enhancement}\left(F_{sp}+F_{adp}\right),$$

(14) $$F_{out}=F+F_{enh}.$$

Through the combination of spatial attention, channel-wise adaptive weighting, and residual feature refinement, RARM effectively recovers subtle details in compressed frames and provides higher-quality representations for subsequent video enhancement and downstream analysis tasks.

### 3.6. Loss Function

To jointly optimize perceptual reconstruction quality and downstream machine vision utility, the proposed framework adopts a hybrid optimization objective that combines pixel-level structural fidelity and texture-aware feature consistency. The overall loss function is formulated as a weighted combination of the Charbonnier loss and the texture loss, where the parameter β controls the relative contribution of reconstruction supervision and texture-guided enhancement during optimization.

Charbonnier loss: A robust variant of the $L_{1}$ loss is adopted to preserve pixel-level structural fidelity while reducing sensitivity to outliers and compression noise:

(15) $$L_{\mathrm{Charbonnier}}=\sqrt{∥\hat{I}-I_{\mathrm{gt}}{∥}^{2}+\epsilon^{2}},$$

where ϵ is a small constant (typically $1\times 10^{-3}$).

Texture loss: To further preserve local texture consistency and compression-sensitive structural details, a texture-aware matching loss is introduced to constrain feature similarity in the local representation space:

(16) $$L_{\mathrm{texture}}=-\sum_{i}\log\left(\frac{\exp(1-d_{ij})}{\sum_{k}\exp\left(1-d_{ik}\right)}\right),$$

where $d_{ij}$ denotes the normalized feature distance between feature representations *i* and *j*.

Overall loss:

(17) $$L_{\mathrm{total}}=\beta L_{\mathrm{Charbonnier}}+\left(1-\beta \right)L_{\mathrm{texture}}.$$

In this work, we set β=0.8 for the Charbonnier loss and 1−β=0.2 for the texture loss. The higher weight on the Charbonnier loss helps maintain stable pixel-level reconstruction quality, while the texture loss contributes to preserving texture and semantic-related information beneficial for downstream machine vision tasks. To further validate the influence of different β settings, additional sensitivity analysis experiments are provided in the ablation study section. Experimental results under multiple β configurations demonstrate that β=0.8 achieves the most stable overall performance across both visual reconstruction quality and downstream machine vision evaluation.

## 4. Experiments

### 4.1. Experimental Setup

We conduct our experiments on the MFQEV2 dataset, which contains 160 high-quality videos sourced from Xiph.org, VQEG, and JCT-VC. The dataset is divided into 106 videos for training and 18 videos for testing. To generate compressed inputs, we process all videos using the H.265 reference encoder (HM16.5) with Low-Delay P (LDP) configuration and a fixed QP value of 37.

The proposed method is implemented using the PyTorch framework, with the experiments conducted in Python 3.7 and PyTorch 1.6.0 on Ubuntu 16.04 with an NVIDIA RTX 3090 GPU.

During training, we prepare the dataset by randomly cropping 128 × 128 patches from raw videos, compressed videos, and their associated coding priors to create training samples. Each video clip contains 7 frames, and we use a batch size of 32.

For optimization, we employ the Adam optimizer with hyperparameters $\beta_{1}=0.9$ and $\beta_{2}=0.999$, and set the initial learning rate to $1\times 10^{-4}$.

### 4.2. Experimental Results for Human Vision

To verify the validity and superiority of the proposed method, we evaluate the enhancement of human vision quality in compressed video using the PSNR (peak signal-to-noise ratio) as the primary metric. As shown in Table 1, our method outperforms all existing approaches. For a comprehensive comparison, we selected eleven state-of-the-art enhancement networks for compressed videos, conducting experiments at a quantization parameter (QP) of 37.

Table 2 presents the comparative results at QP = 42, further validating the effectiveness of our proposed method. In comparison with nine related methods including our previous work, our method consistently outperforms all competing methods across all video classes, achieving superior ΔPSNR gains. These results not only demonstrate the robustness of our approach under stronger-compression conditions but also corroborate the validity of our experimental settings.

Table 3 presents a detailed quantitative evaluation of our proposed method against the STDF baseline across a diverse set of video sequences from the standard classes B, C, D, and E. Four widely used metrics are reported: PSNR gain (ΔPSNR), LPIPS, and SSIM, where ↑ indicates higher values are better, and ↓ indicates lower values are better. As shown, our method achieves significant improvements in perceptual quality, with lower LPIPS scores, and higher SSIM scores in nearly all sequences. For instance, in sequences such as Johnny and FourPeople, our method shows marked perceptual gains with LPIPS values of 0.1757 and 0.1590, respectively, compared to STDF’s 0.1766 and 0.1595. On average, our method achieves a PSNR improvement of 0.8811 dB and notably reduces the LPIPS score while improving SSIM, demonstrating the robustness and effectiveness of our video enhancement strategy across varying content and motion complexity.

**Figure 3:**
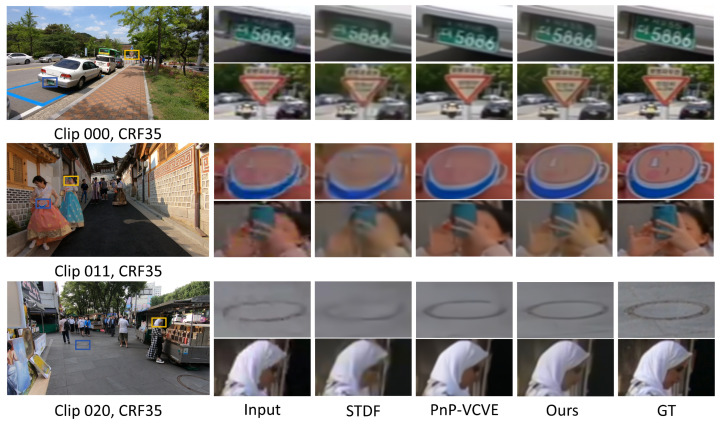
Qualitative results on compressed video quality enhancement for Clip000, 011, and 020 from the REDS test set under CRF35 compression.

To validate the effectiveness of our method, we compare it with recent approaches under different compression settings (previously reported as QP) and datasets. The results of compressed video quality enhancement are shown in Table 4. As shown in Table 4, we compare with representative methods in compressed video enhancement, including MFQE 2.0 [23], STDF [24], S2SVR [47], Metabit [28], and PnP-VCVE [48]. The results of the compressed video quality enhancement are shown in Table 4, evaluated on the REDS4 dataset [49] using the PSNR and SSIM metrics (higher values indicate better quality). For each method, we report the model complexity (parameters and FLOPs) and inference speed (FPS) to provide a comprehensive comparison of both performance and efficiency. The proposed method achieves consistently high PSNR and SSIM values across different compression levels (CRF15, CRF25, CRF35), demonstrating its ability to maintain high visual quality while keeping computational cost and inference time within a reasonable range. Compared with existing approaches, our method exhibits more stable improvements under low, medium, and high compression levels, particularly at high compression (CRF35), where it effectively restores fine textures and details. Figure 3 presents qualitative comparisons on Clip000, 011, and 020 from the REDS test set under CRF35 compression. As shown in the zoomed-in regions, the compressed input suffers from severe compression artifacts, blurred object boundaries, and noticeable texture degradation. Compared with existing enhancement methods such as STDF and PnP-VCVE, the proposed framework produces clearer structural details and more consistent texture restoration while effectively suppressing compression-induced distortions.

**Table 1:** Objective comparison of our method with eleven competing methods under the LDP configuration at QP = 37. Human vision quality enhancement is evaluated using ΔPSNR. The best results are highlighted in red, while the second-best results are highlighted in blue.

		ΔPSNR											
Configuration Class	Sequence	AR-CNN [50]	DnCNN [51]	Li et al. [52]	DCAD [53]	DS-CNN [21]	MFQE 1.0 [22]	MFQE 2.0 [23]	PWSTQ [25]	MRDN [27]	FastCNN [26]	STDF [24]	Ours
B	BQTerrace	0.20	0.20	0.25	0.28	0.26	0.27	0.40	0.43	0.55	0.61	0.605	0.650
	BasketballDrive	0.23	0.25	0.30	0.31	0.28	0.41	0.47	0.49	0.71	0.84	0.736	0.833
	Cactus	0.19	0.20	0.23	0.26	0.24	0.44	0.50	0.60	0.67	0.80	0.710	0.774
	Kimono	0.22	0.24	0.28	0.28	0.25	0.50	0.55	0.57	0.82	1.00	0.861	0.965
	ParkScene	0.14	0.14	0.15	0.16	0.15	0.39	0.46	0.47	0.60	0.62	0.558	0.625
C	BQMall	0.28	0.28	0.33	0.34	0.33	0.51	0.62	0.73	0.90	0.99	0.924	1.065
	BasketballDrill	0.25	0.33	0.38	0.39	0.35	0.48	0.58	0.59	0.73	0.83	0.769	0.866
	PartyScene	0.14	0.14	0.13	0.16	0.17	0.22	0.36	0.51	0.55	0.59	0.60	0.667
D	BQSquare	0.08	0.13	0.09	0.20	0.20	−0.01	0.34	0.69	0.75	0.77	0.809	0.963
	BasketballPass	0.26	0.31	0.34	0.35	0.34	0.63	0.73	0.80	0.98	1.05	0.964	1.093
	BlowingBubbles	0.16	0.18	0.21	0.22	0.23	0.39	0.53	0.62	0.66	0.69	0.670	0.758
	RaceHorses	0.27	0.31	0.33	0.34	0.32	0.51	0.59	0.61	0.83	0.85	0.735	0.833
E	Johnny	0.25	0.32	0.40	0.41	0.38	0.55	0.60	0.69	1.12	0.88	0.786	0.910
	FourPeople	0.37	0.39	0.45	0.51	0.46	0.66	0.73	0.95	0.94	1.05	0.944	1.075
	KristenAndSara	0.41	0.42	0.49	0.52	0.48	0.66	0.75	0.89	0.95	1.13	1.008	1.139
Average ΔPSNR		0.23	0.256	0.2907	0.3153	0.296	0.4407	0.5473	0.6427	0.78	0.85	0.7786	0.8811

**Table 2:** Objective comparison of our method with nine competing methods under the LDP configuration at QP = 42. Human vision quality enhancement is evaluated using ΔPSNR. The best results are highlighted in red, while the second-best results are highlighted in blue.

		ΔPSNR									
Configuration Class	Sequence	Li et al. [52]	DS-CNN [21]	MFQE 2.0 [23]	CVRGAN [54]	MW-GAN [55]	MW-GAN+ [42]	PWSTQ [25]	STDF [24]	Ours_ICIP [31]	Ours
B	BQTerrace	0.290	0.302	0.489	0.369	0.301	0.582	0.53	0.564	0.669	0.681
	BasketballDrive	0.307	0.301	0.561	0.480	0.428	0.839	0.55	0.676	0.793	0.825
	Cactus	0.271	0.262	0.548	0.340	0.333	0.782	0.62	0.609	0.739	0.742
	Kimono	0.285	0.292	0.615	0.286	0.277	0.908	0.58	0.701	0.820	0.844
	ParkScene	0.197	0.199	0.399	0.212	0.302	0.426	0.38	0.415	0.496	0.504
C	BQMall	0.325	0.324	0.638	0.393	0.380	0.748	0.69	0.714	0.853	0.879
	BasketballDrill	0.302	0.345	0.828	0.444	0.535	0.797	0.60	0.702	0.851	0.867
	PartyScene	0.186	0.202	0.440	0.164	0.209	0.470	0.47	0.432	0.504	0.514
D	BQSquare	0.095	0.153	0.397	-0.027	0.393	0.331	0.63	0.613	0.749	0.805
	BasketballPass	0.286	0.284	0.709	0.353	0.363	0.870	0.72	0.745	0.903	0.911
	BlowingBubbles	0.182	0.191	0.461	0.199	0.225	0.423	0.45	0.444	0.509	0.516
	RaceHorses	0.302	0.303	0.594	0.251	0.331	0.634	0.60	0.643	0.763	0.776
E	Johnny	0.432	0.404	0.680	0.531	0.663	0.827	0.70	0.654	0.799	0.813
	FourPeople	0.451	0.473	0.704	0.542	0.578	0.829	0.79	0.655	0.836	0.823
	KristenAndSara	0.459	0.423	0.762	0.582	0.692	0.806	0.84	0.807	0.981	0.999
Average ΔPSNR		0.2913	0.2972	0.5883	0.3413	0.4007	0.6848	0.61	0.6249	0.7510	0.7666

**Table 3:** Quantitative comparison of video quality enhancement methods on different sequences from configuration classes B, C, D, and E. Metrics include ΔPSNR (↑), LPIPS (↓), and SSIM (↑). Our method consistently outperforms the baseline STDF across all metrics, especially in perceptual quality indicators such as LPIPS and SSIM. Bold values indicate the best performance.

Configuration Class	Sequence	Ours			STDF		
		ΔPSNR ↑	LPIPS ↓	SSIM ↑	ΔPSNR ↑	LPIPS ↓	SSIM ↑
B	BQTerrace	**0.650**	**0.2782**	**0.8170**	0.605	0.2783	0.7923
	BasketballDrive	**0.833**	**0.3151**	**0.8367**	0.736	0.3184	0.8176
	Cactus	**0.774**	**0.325**	**0.7954**	0.711	0.3251	0.7725
	Kimono	**0.965**	0.3429	**0.8651**	0.861	**0.3379**	0.8596
	ParkScene	**0.625**	0.3812	**0.7911**	0.557	**0.3795**	0.7822
C	BQMall	**1.065**	**0.1972**	**0.8577**	0.924	0.2033	0.8447
	BasketballDrill	**0.866**	**0.2303**	**0.8074**	0.769	0.2358	0.7907
	PartyScene	**0.667**	**0.1895**	**0.7846**	0.6	0.196	0.77
D	BQSquare	**0.963**	**0.1716**	**0.8244**	0.809	0.1748	0.7925
	BasketballPass	**1.093**	**0.2075**	**0.8132**	0.964	0.2136	0.7925
	BlowingBubbles	**0.758**	**0.2108**	**0.7518**	0.67	0.2192	0.7422
	RaceHorses	**0.833**	**0.2253**	**0.7503**	0.735	0.2284	0.7391
E	Johnny	**0.910**	**0.1757**	**0.9205**	0.786	0.1766	0.9063
	FourPeople	**1.075**	**0.1590**	**0.9204**	0.944	0.1595	0.9110
	KristenAndSara	**1.139**	**0.1653**	**0.9312**	1.008	0.1646	0.9217
Average		**0.8811**	**0.2383**	**0.8311**	0.7786	0.2407	0.8157

**Table 4:** Comparison of different video enhancement methods on the REDS dataset with CRF15, CRF25, and CRF35 compressed videos. The table reports the number of parameters, FLOPs, FPS, and the quantitative quality metrics PSNR and SSIM. Our method achieves the best balance between efficiency and performance across all compression levels. Bold values indicate the best performance, while underlined values indicate the second-best performance.

Method	Param/M	FLOPs/G	FPS	CRF15		CRF25		CRF35	
				PSNR	SSIM	PSNR	SSIM	PSNR	SSIM
MFQE 2.0 [23]	1.64	51	19	40.95	0.9806	34.83	0.9378	29.22	0.8256
STDF [24]	1.27	45	26	41.15	0.9793	35.23	0.9398	29.74	0.8359
S2SRV [47]	1.43	294	-	41.96	0.9834	35.61	0.9445	29.87	0.8391
Metabit [28]	1.6	92	42	41.04	0.9785	34.92	0.9363	29.25	0.8238
PnP-VCVE [48]	4.56	47	28	**42.22**	**0.9842**	**35.90**	**0.9468**	30.17	0.8471
Ours	2.34	51	30	41.98	0.9832	35.69	0.9450	**30.78**	**0.8494**

### 4.3. Experimental Results for Machine Vision

**Figure 4:**
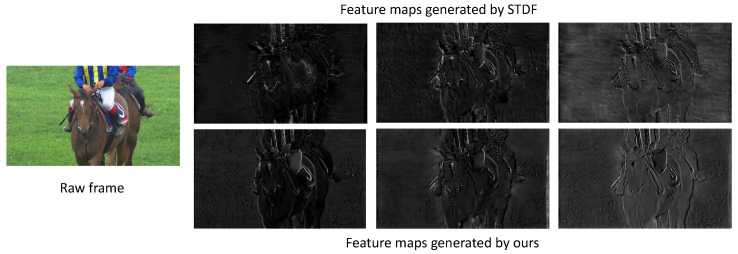
Visualization of feature maps. Given a raw input frame (left), the top row shows feature maps generated by STDF, while the bottom row presents those generated by our method. Our approach yields more distinct and sharper feature representations.

Figure 4 illustrates a visual comparison between the feature maps generated by the baseline STDF model and our proposed method. As shown, given the same raw input frame (left), the feature maps produced by STDF (top right) exhibit relatively blurry and less-discriminative structures. In contrast, our method (bottom right) captures more prominent object boundaries and motion cues, demonstrating better feature expressiveness. This highlights the effectiveness of our architecture in preserving structural details under compression noise.

**Figure 5:**
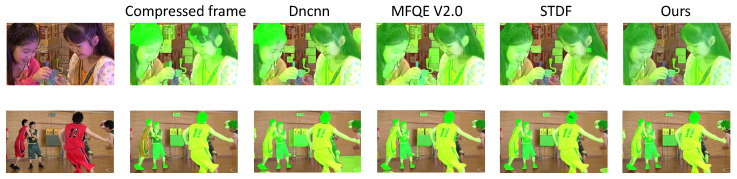
Visual comparison of segmentation results on compressed frames processed by different enhancement methods using FastSAM.

Figure 5 shows segmentation outputs produced by Fast Segment Anything (FastSAM) on compressed video frames enhanced by different methods. From left to right, the columns represent: the original compressed frame, DnCNN, MFQE V2.0, STDF, and our proposed method. Green masks represent the segmented regions. It can be observed that our method yields more complete and accurate segmentation, especially in maintaining boundary integrity and object details, demonstrating better robustness under compression artifacts.

For the video object segmentation (VOS) task, we evaluate our approach using the DAVIS-17 validation dataset [56] and report results with three standard metrics: region similarity (J, measured by average IoU), boundary accuracy (F score), and their average (Avg). For baselines, we select two representative methods: STCN [57] and QDMN [58]. Higher scores across all metrics correspond to better segmentation performance. The experiments were conducted under two different video compression quality settings, QP37 and QP42, to comprehensively validate the robustness of the method. The quantitative results of the VOS task are shown as in Table 5.

Under the STCN framework, our proposed method demonstrates significant advantages under both compression levels. In the QP37 setting, our approach achieves leading results with J = 84.81, F = 97.65, and Avg = 91.23, outperforming the original STCN (Avg = 89.63) as well as its combinations with DnCNN, MFQE 2.0, and STDF. This improvement remains consistent with the higher compression of QP42, where the proposed method reaches an Avg = 88.72, exceeding the original STCN performance by more than 1.6 points, indicating strong resilience to compression artifacts. A similar trend is observed for the QDMN framework. In particular, under QP42 conditions our approach achieves J = 77.87, F = 95.91, and Avg = 86.89, showing a notable improvement over the baseline QDMN (Avg = 83.99) and outperforming all other enhancement strategies. In the QP37 setting, our method also achieves the highest F score (97.24) and overall score (Avg = 89.71), further confirming its consistency and effectiveness under different bitrate conditions.

**Figure 6:**
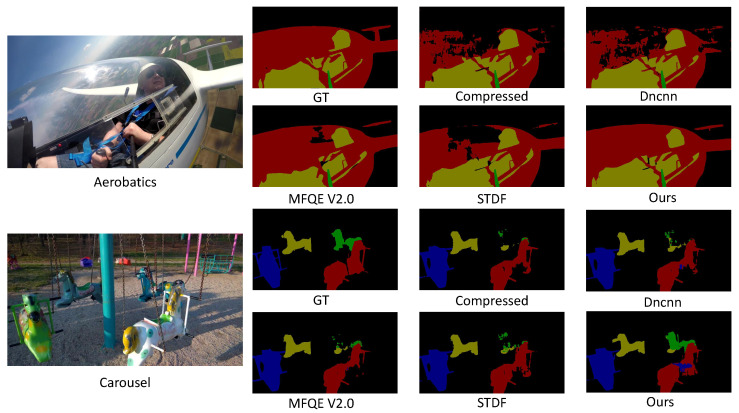
Qualitative results of VOS using STCN. As can be seen, directly performing VOS on compressed inputs leads to inaccurate masks, but our method effectively improves the accuracy, especially for the regions of irregular shapes.

Figure 6 presents the visualization results of the VOS task using STCN. The compressed image input clearly leads to increased errors in the segmentation mask, particularly around object contours.

In the aerobatics scene, a comparison between GT and the compressed input reveals that compression causes confusion between the background and foreground, especially in the region between the figure and the cockpit. While traditional methods such as DnCNN and MFQE 2.0 show some improvement, they still exhibit inaccuracies in details. In contrast, our method recovers clearer contour structures, and the segmented regions align more closely with GT. In the carousel scene, multiple colored objects are situated against a complex background. Compression results in severe boundary blurring of occluded objects (e.g., the green horse). Both DnCNN and MFQE 2.0 still struggle to distinguish certain areas. Although STDF shows some enhancement, confusion remains. Our method demonstrates superior performance in shape preservation, occlusion recovery, and edge continuity, noticeably outperforming other approaches.

Table 6 presents a comparison of the mean average precision (mAP) for object detection across three video categories (C, D, and E) under four quantization parameter (QP) settings (QP = 37, 42, 47, 51), using four different approaches: without compensation (w/o), STDF, our earlier work, and the proposed method. The table also reports the performance gap between our method and previous approaches, along with the average improvement at the bottom.

As shown in Table 6, our method achieves the best detection performance across all categories and compression levels. Notably, under high-compression conditions (QP = 51), it attains an average mAP improvement of 6.08%, demonstrating its strong effectiveness and practical value in object detection for compressed videos.

### 4.4. Ablation Study

To verify the effectiveness of each proposed module (HSFS, RARM, and TGM), we conduct a comprehensive ablation study, as shown in Table 7 and Table 8. We adopt a progressive module-addition strategy for the ablation study, in which the proposed modules (HSFS, RAR-QE, and TGM) are incrementally integrated into the base network, and the resulting performance gains are reported. This design allows us to directly observe the cumulative effects of each module as the network evolves.

In the video enhancement task, as shown in Table 7, the progressive integration of modules consistently improves performance across all metrics. When only the HSFS module is used, the model achieves a ΔPSNR of 0.864, LPIPS of 0.2383, and SSIM of 0.8171. After incorporating the RARM module, ΔPSNR and SSIM both improve significantly, indicating that RARM enhances spatial feature modeling. When the TGM module is further added, all metrics reach their best values: ΔPSNR increases to 0.881, LPIPS decreases to 0.2383, and SSIM improves to 0.8311. This confirms that the combination of the three modules works synergistically to boost video quality.

In the video object detection task, as shown in Table 8, we evaluate the mAP improvement under different quantization parameters. Using only the HSFS module yields the lowest performance gain. Adding the RARM module leads to better improvements across all QPs. With all three modules integrated, the model achieves the highest gains: +1.47, +4.13, +4.43, and +6.08 mAP at QP32, QP42, QP47, and QP51, respectively. This demonstrates that the TGM module plays a crucial role in temporal modeling.

**Figure 7:**
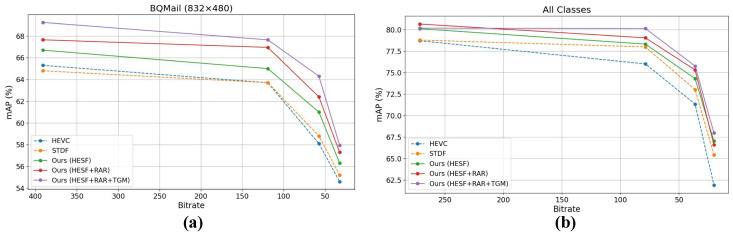
Rate-distortion performance. Our method outperforms the other approaches at various bitrates. (**a**) Rate-distortion performance on the BQMall sequence (832 × 480); (**b**) Rate-distortion performance averaged over all classes.

Figure 7 presents the curve charts for object detection, which include two line graphs showing the performance of multiple methods at different compression ratios (bitrates) for the “BQMail (832 × 480)” sequence and “All Classes”, respectively. In the subfigure for BQMail (832 × 480), our final method (purple curve) consistently achieves the highest mAP values across all bitrates, with its performance advantage being particularly notable at higher compression ratios. In the second subfigure, the methods are evaluated on a broader range of data categories. The overall trend remains consistent: as the bitrate decreases, the mAP of all methods declines, but our proposed method still maintains leading performance across all bitrate segments. In summary, the step-by-step introduction of HSFS, RARM, and TGM leads to significant performance improvements. The final model, which incorporates all three components, consistently achieves the best results in both the video enhancement and object detection tasks, showing the complementary strengths of these modules.

To further investigate the influence of the parameter β in the proposed hybrid loss function, we conduct additional experiments under different β settings, including β=0, β=0.6, β=0.8, and β=1.0, as summarized in Table 9. In our framework, β controls the relative contribution between the Charbonnier reconstruction loss and the texture-guided enhancement loss during optimization.

When β=0, the optimization is fully dominated by the texture-guided enhancement loss without reconstruction supervision. Although the VOS performance remains competitive, the PSNR and SSIM values decrease significantly, indicating that texture-guided enhancement alone is insufficient for preserving stable pixel-level reconstruction quality. As β increases, the reconstruction quality is consistently improved, demonstrating the importance of reconstruction supervision for visual restoration. On the other hand, simply increasing β does not continuously improve downstream machine vision performance. Although β=1.0 achieves reconstruction quality comparable to β=0.8, its J (IoU) and F-score metrics decrease. This suggests that relying only on reconstruction supervision may weaken the preservation of semantic-related information beneficial for downstream tasks.

Overall, β=0.8 achieves the most stable overall quantitative performance, obtaining the highest PSNR, SSIM, and competitive VOS results simultaneously. These results demonstrate that combining reconstruction supervision with texture-guided enhancement is beneficial for both visual quality restoration and downstream machine vision performance.

## 5. Discussion

**Figure 8:**
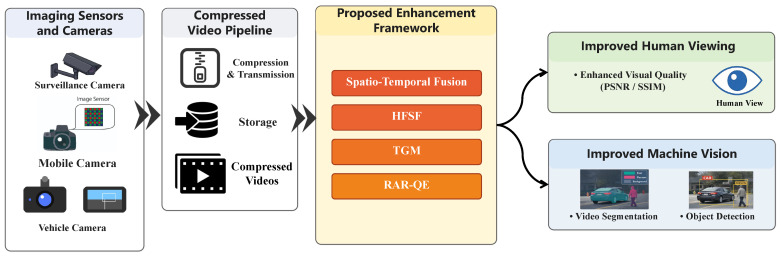
Application of the proposed framework in image sensor-based systems.

Video streams captured by imaging sensors constitute a major source of visual data in modern multimedia and vision systems. In many practical applications, these video streams are compressed to reduce transmission and storage costs, especially in scenarios with limited bandwidth, restricted memory, or constrained computational resources. While compression is essential for efficient deployment, it inevitably introduces artifacts that affect not only human perceptual quality but also the effectiveness of downstream machine vision tasks. Therefore, compressed video enhancement is of particular importance in image sensor-based systems that rely on the same visual data for both human observation and machine interpretation. Figure 8 shows the practical role of the proposed framework in image sensor-based systems. In such pipelines, video captured by imaging sensors is often compressed for transmission and storage, while our method can be inserted after decoding as a post-processing module to enhance both perceptual quality and machine vision utility.

From this perspective, the proposed framework provides a useful solution for practical sensing and imaging applications. By jointly considering perceptual restoration and semantic preservation, the method improves the quality of compressed video in a manner that is beneficial to both human-oriented and machine-oriented usage. This property is valuable in applications such as intelligent surveillance, autonomous systems, mobile imaging, and edge vision, where compressed video generated by cameras and imaging sensors is frequently used for subsequent visual analysis. The experimental improvements in PSNR, SSIM, object detection, and video object segmentation suggest that the proposed approach can effectively improve the utility of compressed sensor-generated visual data.

An additional practical merit of the proposed framework is that it can be integrated into existing multimedia pipelines without changing the front-end sensing hardware or standard video coding procedures. As a decoder-side or post-processing enhancement module, it can be deployed after video reconstruction to mitigate quality degradation while preserving compatibility with existing imaging and communication systems. This makes the framework attractive for real-world use cases in which upgrading hardware or modifying codec standards is undesirable.

Nevertheless, some limitations should also be noted. The current evaluation is mainly conducted on benchmark compressed video datasets, and further validation under broader real-world sensing conditions would be valuable. Moreover, practical deployment in resource-constrained platforms may require additional optimization in model efficiency and inference latency. Future work may therefore explore lighter implementations, scenario-adaptive enhancement strategies, and broader evaluation on sensor-driven applications with varying acquisition characteristics.

## 6. Conclusions

This paper presents a unified framework for enhancing compressed video quality, designed to jointly optimize both human visual perception and machine analysis tasks. By integrating a novel Texture-Guided Model with a Spatio-Temporal Fusion Module, High-Frequency Semantic Fusion module, and Refined Attention Residual Quality Enhancement module through a consistent multi-fusion strategy, our approach effectively addresses the limitations of existing methods. Extensive experimental results demonstrate that our framework achieves strong performance across multiple domains: it significantly improves video quality metrics (PSNR/SSIM) for human viewing, while simultaneously boosting performance in machine vision tasks including object detection (mAP) and video object segmentation (IoU and boundary similarity). This work provides an effective solution for applications requiring dual optimization, such as autonomous driving and video surveillance systems, where both visual quality and machine task performance are critical. Since these applications commonly rely on video data captured by imaging Z, the proposed framework also offers a practical post-processing solution for improving compressed video quality in image sensor-based multimedia systems.

## Figures and Tables

**Table 5:** Quantitative results of VOS, where the best and second-best results are highlighted with bold and underlined. The symbol ↑ indicates that higher values are better.

Method	QP37			QP42		
	J ↑	F ↑	Avg. ↑	J ↑	F ↑	Avg. ↑
STCN	82.62	96.69	89.63	78.21	95.87	87.04
+DnCNN [51]	82.55	96.48	89.5	75.78	92.59	85.23
+MFQE 2.0 [23]	82.85	96.18	89.52	78.38	95.95	87.17
+STDF [24]	83.19	97.19	90.19	78.62	95.59	87.1
+Ours	**84.81**	**97.65**	**91.23**	**80.92**	**96.21**	**88.72**
QDMN	80.18	96.37	88.28	74.06	93.93	83.99
+DnCNN [51]	79.48	96.09	87.78	73.31	94.33	83.82
+MFQE 2.0 [23]	80.02	96.31	88.17	75.39	95.62	85.51
+STDF [24]	80.74	96.7	88.72	75.28	94.71	84.99
+Ours	**82.18**	**97.24**	**89.71**	**77.87**	**95.91**	**86.89**

**Table 6:** Comparison results of the detection accuracy with STDF and our previous work at the same bitrate via YOLOv8. The symbol ↑ indicates that higher values are better. The best performance is highlighted in bold.

Class	Method	QP			
		37	42	47	51
C	w/o	75.50	71.63	65.30	59.70
	STDF [24]	76.03	74.00	68.63	61.03
	Ours_ICIP [31]	75.90	73.40	70.64	65.37
	Ours	**78.79**	**77.79**	**72.99**	**66.69**
	Gap	+3.29	+6.16	+7.69	+6.99
D	w/o	69.85	66.68	62.40	50.80
	STDF [24]	69.08	68.00	62.58	52.80
	Ours_ICIP [31]	**72.83**	69.60	64.13	56.03
	Ours	70.48	**70.46**	**65.30**	**57.15**
	Gap	+0.63	+3.78	+2.90	+6.35
E	w/o	90.80	89.76	86.33	75.23
	STDF [24]	91.30	92.00	87.90	**82.46**
	Ours_ICIP [31]	**91.63**	92.00	88.13	79.70
	Ours	91.30	**92.20**	**89.02**	80.13
	Gap	+0.50	+2.44	+2.69	+4.90
Avg. mAP (%) ↑		+1.47	+4.13	+4.43	+6.08

**Table 7:** Ablation study of the proposed modules (HSFS, RARM, and TGM) on video enhancement performance. Ablation study of the proposed modules (HSFS, RARM, and TGM) on video object detection task. The symbol ✓ indicates that the corresponding module is included in the model. The symbol ↑ indicates that higher values are better, while ↓ indicates that lower values are better. The best performance is highlighted in bold.

Model			ΔPSNR ↑	LPIPS ↓	SSIM ↑
HSFS	RARM	TGM			
			0.7786	0.2407	0.8157
✓			0.864	0.2383	0.8171
✓	✓		0.874	0.2393	0.8275
✓	✓	✓	**0.881**	**0.2383**	**0.8311**

**Table 8:** Ablation study of the proposed modules (HSFS, RARM, and TGM) on video object detection task. The symbol ✓ indicates that the corresponding module is included in the model. The symbol ↑ indicates that higher values are better. The best performance under each QP setting is highlighted in bold.

Model			mAP ↑			
HSFS	RARM	TGM	QP32	QP42	QP47	QP51
			+0.08	+1.98	+1.69	+3.52
✓			+1.4	+2.31	+2.96	+5.12
✓	✓		**+1.95**	+3.04	+3.98	+4.7
✓	✓	✓	+1.47	**+4.13**	**+4.43**	**+6.08**

**Table 9:** Sensitivity analysis of the parameter β on the DAVIS-17 validation dataset. The results are evaluated using checkpoints obtained after 200k training iterations. Human vision quality is measured by PSNR and SSIM, while machine vision performance is evaluated on the VOS task using the QDMN framework with J (IoU) and F-score metrics. The symbol ↑ indicates that higher values are better. The best performance is highlighted in bold.

	Visual Metrics		Machine Vision	
β	ΔPSNR ↑	SSIM ↑	J (IoU) ↑	F-Score ↑
0	0.0699	0.8286	**69.260**	90.72
0.6	0.3550	0.8415	67.776	**91.14**
0.8	**0.4581**	**0.8434**	68.250	90.77
1.0	0.4442	0.8431	67.110	90.58

## Data Availability

The data presented in this study are available upon request from the corresponding author.
